# Investigations into metabolic properties and selected nutritional metabolic byproducts of different non-*Saccharomyces* yeast strains when producing nonalcoholic beer

**DOI:** 10.1093/femsyr/foac042

**Published:** 2022-08-25

**Authors:** Yvonne Methner, Nadine Weber, Oliver Kunz, Martin Zarnkow, Michael Rychlik, Mathias Hutzler, Fritz Jacob

**Affiliations:** Research Center Weihenstephan for Brewing and Food Quality, Technical University of Munich, Alte Akademie 3, 85354 Freising, Germany; Chair of Analytical Food Chemistry, Technical University of Munich, Maximus-von-Imhof-Forum 2, 85354 Freising, Germany; Research Center Weihenstephan for Brewing and Food Quality, Technical University of Munich, Alte Akademie 3, 85354 Freising, Germany; Research Center Weihenstephan for Brewing and Food Quality, Technical University of Munich, Alte Akademie 3, 85354 Freising, Germany; Chair of Analytical Food Chemistry, Technical University of Munich, Maximus-von-Imhof-Forum 2, 85354 Freising, Germany; Centre for Nutrition and Food Sciences, Queensland Alliance for Agriculture and Food Innovation, The University of Queensland, 306 Carmody Road, St Lucia QLD 4072, Australia; Research Center Weihenstephan for Brewing and Food Quality, Technical University of Munich, Alte Akademie 3, 85354 Freising, Germany; Research Center Weihenstephan for Brewing and Food Quality, Technical University of Munich, Alte Akademie 3, 85354 Freising, Germany

**Keywords:** non-*Saccharomyces* yeasts, fermentation, nonalcoholic beers, vitamin B, biogenic amines, stress tolerance tests

## Abstract

Nonalcoholic beers are becoming increasingly popular, in part due to consumers’ awareness of a healthier lifestyle. Additionally, consumers are demanding diversification in the product range, which can be offered by producing nonalcoholic beers using non-*Saccharomyces* yeasts for fermentation to create a wide variety of flavors. So far, little is known about the nutritionally relevant byproducts that these yeasts release during wort fermentation and whether these yeasts can be considered safe for food fermentations. To gain insights into this, the B vitamins of four different nonalcoholic beers fermented with the yeast species *Saccharomycodes ludwigii, Cyberlindnera saturnus* (two strains), and *Kluyveromyces marxianus* were analyzed. Furthermore, a total of 16 beers fermented with different non-*Saccharomyces* yeast strains were analyzed for biogenic amines. Additionally, stress tolerance tests were performed at 37°C and in synthetic human gastric juice *in vitro*. B vitamins were found in the four nonalcoholic beers in nutritionally relevant amounts so they could serve as a supplement for a balanced diet. Biogenic amines remained below the limit of determination in all 16 beers, and thus likely had no influence, while the stress tolerance tests gave a first indication that seven yeast strains could possibly tolerate the human gastric juice milieu.

## Teaser

A total of 16 investigated non-*Saccharomyces* yeast strains suitable for producing nonalcoholic beers did not synthesize significant amounts of biogenic amines, while seven yeast strains were viable at 37°C in synthetic gastric juice milieu and four nonalcoholic beers fermented with selected non-*Saccharomyces* yeast strains contained nutritionally relevant concentrations of B vitamins.

## Introduction

Nonalcoholic beers are on trend. Market forecasts predict growth and studies also indicate that this product sector has grown in recent years (Bellut and Arendt 2019, Ahuja and Rawat 2020, Kokole et al. 2021). There are various reasons for this development, ranging from an increasingly healthier lifestyle to religious restrictions and consumer preferences (Salanță et al. 2020). One option for producing nonalcoholic beers is to use maltose- and maltotriose-negative non-*Saccharomyces* yeasts, which have only a low fermentative activity based on this property. Accordingly, these kind of yeast strains offer a simple way to produce nonalcoholic or low-alcohol beers. In a recently published study by Methner et al. (2022), 16 different non-*Saccharomyces* yeast strains were investigated for their suitability to produce nonalcoholic beers with novel flavor profiles. Promising flavor profiles emerged and their suitability for the production of nonalcoholic beers was confirmed. An increasing number of maltose-negative yeast strains have been investigated in recent years to produce nonalcoholic beers with pronounced flavor diversity. However, not much is known about the nutritional properties of these beers such as B vitamins or biogenic amines (BAs).

The B vitamin group is composed of eight different water-soluble vitamins. These eight B vitamins act as coenzymes in numerous catabolic and anabolic reactions and are indispensable for optimal physiological and neurological functions in human metabolism. Kennedy summarized the key mechanisms, Recommended Daily Allowance (RDA) and efficacy (Kennedy 2016). His research reveals that thiamine (B_1_), riboflavin (B_2_), niacin (B_3_), pantothenic acid (B_5_), pyridoxine (B_6_), biotin (B_7_), and folate (B_9_) are mainly synthesized by plants, whereas cobalamin (B_12_) is synthesized by bacteria. Moreover, yeasts may have the ability to synthesize B vitamins. For example, it is known from existing studies that brewer’s spent yeast is rich in vitamins, especially B_3_, B_6_, and B_9_ (Ferreira et al. 2010, Vieira et al. 2016). In this context, *Saccharomyces cerevisiae* was found to produce particularly high amounts of folate during yeast extract production (Jacob et al. 2019). Regular beers produced by using traditional *Saccharomyces* yeasts have already been analyzed for B vitamins, which may be present in sufficient amounts to meet daily requirements (Bamforth 2002). In a study by Mayer et al. (2001), moderate beer consumption was even found to have certain positive health effects due to an increase in folate and vitamin B_12_ intake. Nevertheless, it remains undisputed that ethanol can have a negative impact on health (Marten et al. 2020). Accordingly, nonalcoholic beer could provide positive health benefits, given that B vitamins are present in significant concentrations. Since all eight B vitamins are essential for the human metabolism, they must be consumed through diet. Little is known to date about the vitamin B composition of nonalcoholic beers produced with maltose-negative yeast strains. As the health trend steadily increases (Habschied et al. 2020), the purpose of this study was to screen nonalcoholic beers for B vitamins. A total of four nonalcoholic beers were selected, which were produced with maltose-negative yeasts strains found in a previous study to form particularly fruity flavors during wort fermentation (Methner et al. 2022). These were the yeast strains *Cyberlindnera saturnus* C. sat 247 and C. sat CSa1 as well as *Kluyveromyces marxianus* K. mar 653. Additionally, a nonalcoholic beer produced with the well-known and commercially used yeast strain *Saccharomycodes ludwigii* S. lud SL17 was chosen, which was also analyzed.

BAs also play a decisive role from a health perspective. For example, they perform important tasks as messenger substances in the neural system. However, intolerance, and thus adverse health effects can occur if certain intake concentrations are exceeded (European Food Safety Authority 2011, Bayerisches Landesamt für Gesundheit und Lebensmittelsicherheit 2020). Moderate amounts of about 50 mg/kg food are considered safe (Loret et al. 2005). Since BAs can be formed during the fermentation process, there are numerous studies on their occurrence in beers, which were summarized in a review by Kalac and Krízek (2003). This states that it is mainly bacterial contamination that leads to BA formation. Nevertheless, Ternes (2007) found that the BAs putrescine, spermine, and spermidine occur naturally in beer, whereas histamine, tyramine, and cadaverine are formed by microbes. Few studies report that yeasts such as *S. cerevisiae* and also several non-*Saccharomyces* yeast strains are capable of synthesizing BAs in a species- or even strain-dependent manner, which is why this formation pathway should also be considered (Caruso et al. 2002, Torrea and Ancín 2002, Granchi et al. 2005, Beneduce et al. 2010, Wang et al. 2021). Similar concentrations of BAs were found in alcoholic as well as alcohol-free beers (Buiatti et al. 1995, Izquierdo-Pulido et al. 1996, Kalac and Krízek 2003). In foods, the highest importance is attributed to the BAs histamine, putrescine, cadaverine, tyramine, tryptamine, phenylethylamine, spermine, and spermidine (Shalaby 1996). In the aforementioned study by Methner et al. (2022), a total of 16 different non-*Saccharomyces* yeast strains turned out to be suitable for producing nonalcoholic beers by means of microbiological manufacturing processes. To date, nothing is known about the yeast species *Cyberlindnera fabianii, Cyberlindnera misumaiensis, C. saturnus, Kazachstania servazzii, Kluyveromyces lactis, K. marxianus, Lachancea kluyveri, Pichia kluyveri*, and *Saccharomycopsis fibuligera* with regard to the formation of BAs. For the yeast species *P. kluyveri*, this assumption has already been made in the literature (Landete et al. 2007, Vicente et al. 2021). Regarding the yeast species *Schizosaccharomyces pombe* and *S. ludwigii*, it is reported from wine fermentations that they can even cause a reduction in BAs (Benito et al. 2015, Ivit et al. 2018). Only for *Torulaspora delbrueckii* it was reported that in winemaking this yeast species might be able to slightly increase BA precursors (Benito 2018). Therefore, it was investigated in this study, whether the 16 specified yeast strains could release the most food-relevant BAs during fermentation.

Besides BAs, there are further factors that can lead to safety concerns in the context of non-*Saccharomyces* yeasts for the production of nonalcoholic beers. Thus, for reasons of precautionary consumer protection, only yeasts that have been granted GRAS (Generally Recognized As Safe) status by the US Food and Drug Administration (FDA) or QPS (Qualified Presumption of Safety) status by the European Food Safety Authority (EFSA) should be used (Steensels and Verstrepen 2014, U.S. Food and Drug Administration 2017). While QPS status mainly considers the safety of the microorganism regardless of application, GRAS status refers to specific food applications. In the USA, microorganisms hold GRAS status if they have been commonly used in food applications before 1958, and thus are known to have a history of safe use (U.S. Food and Drug Administration 2018). EFSA, on the other hand, grants QPS status based on risk assessments of biological agents conducted by the Panel on Biological Hazards (BIOHAZ; EFSA-BIOHAZ Panel 2007, 2021). A more detailed overview of the differences between QPS and GRAS is provided in a review by Laulund et al. (2017). Of the 16 yeast species investigated in this study, only three species besides the reference yeast *S. cerevisiae* have QPS status: *K. lactis, K. marxianus*, and *S. pombe* (Ricci et al. 2018). Unless yeasts can grow at 37°C, potential pathogenicity is largely excluded. However, if yeasts are viable at 37°C, this does not necessarily mean that the yeasts are pathogenic, as there may also be a potential probiotic benefit, as is known for both *S. cerevisiae* var. *boulardii* and *S. cerevisiae* (Gil-Rodriguez 2015, Lara-Hidalgo et al. 2017). However, before probiotic potential can be considered, it must be established that microorganisms can be used without safety concerns. Since few findings in this regard have been found in literature for the 16 yeast strains studied, they were tested to determine whether they could grow at 37°C. In addition, they were subjected to a stress tolerance test in synthetic gastric juice to gain initial insights into their physiological properties.

The aim of this study was, therefore, to examine nonalcoholic beers fermented with selected non-*Saccharomyces* yeast strains for selected BAs and B vitamins in order to make a statement about these selected nutritionally relevant metabolic yeast byproducts. Furthermore, the yeasts were investigated *in vitro* for certain physiologically relevant properties to give an initial indication that they can tolerate conditions in the human gastric system.

## Materials and methods

### Investigated yeast strains

Table 1 lists the yeast strains with the corresponding abbreviations that were investigated in this study. With the exception of the yeast strain C. sat CSa1, which is a proprietary strain of a specific brewery (provided for this study and stored at the Research Center Weihenstephan, TUM), all other yeast strains can be purchased commercially. The collection site from which the yeast strains were obtained is indicated in the footnote of Table 1.

**Table 1.** Yeast species and strain numbers with corresponding abbreviations used in this study.

Yeast strain number	Yeast strain abbreviation	Yeast species
CBS 5640	C. fab 5640	*C. fabianii* ^a^
CBS 5650	C. fab 5650	*C. fabianii* ^a^
TUM 238	C. mis 238	*C. misumaiensis* ^b^
YH837A-3D4	C. mis CM1	*C. misumaiensis* ^b^
TUM 247	C. sat 247	*C. saturnus* ^b^
CBS 4549	C. sat 4549	*C. saturnus* ^a^
YHMH22AA-3H1	C. sat CSa1	*C. saturnus* ^c^
YHMH47B-3C4	K. ser 3C4	*K. servazzii* ^b^
TUM G9K	K. lac G9K	*K. lactis* ^b^
TUM 653	K. mar 653	*K. marxianus* ^b^
CBS 3082T	L. klu 3082	*L. kluyveri* ^a^
YHAK1A-3I1	P. klu PK1	*P. kluyveri* ^b^
TUM 68	S. cer 68	*S. cerevisiae* ^b^
TUM SL17	S. lud SL17	*S. ludwigii* ^b^
PI S 6; Lu27	S. fib Lu27	*S. fibuligera* ^b^
TUM G10S	S. pom G10S	*S. pombe* ^b^
YH824A-1I6	T. del 1I6	*T. delbrueckii* ^b^

a Westerdijk Fungal Biodiversity Institute, Utrecht, The Netherlands; ^b^Research Center Weihenstephan (BLQ), Freising, Germany; and ^c^Private collection.

### Determination of vitamin B

A total of four selected non-*Saccharomyces* yeast strains from Table 1, namely C. sat 247, C. sat CSa1, K. mar 653, and S. lud SL17, were inoculated from wort slant agars under sterile conditions into 500 ml flasks each containing 250 ml of unhopped wort (7.0°P, pH 5.5) that was prepared from malt extract (Weyermann®, Bamberg, Germany). After 72 h propagation at 20°C on a WiseShake orbital shaker (Witeg Labortechnik GmbH, Wertheim, Germany) at 80 rpm, the yeast suspensions were transferred to sterile 2500 ml flasks containing 1800 ml of similar unhopped wort and propagated for an additional 72 h. After propagation, the cell counts were determined using the Cellometer® Vision (Nexcelom Bioscience LLC, Lawrence, MA, USA).

The four yeast strains were selected as they were able to form particularly positive flavor properties during fermentation (Methner et al. 2022), and were then used to produce nonalcoholic beers. In small-scale fermentations performed in triplicate, 1800 ml unhopped wort (7.0°P, pH 5.5) in 2000 ml sterile Duran glass bottles (Schott AG, Mainz, Germany) were pitched each at 15 × 10^6^ cells/ml (± σ = 1 × 10^6^ cells/ml) and closed with glass fermentation airlocks on top. To produce the wort, malt extract (Weyermann®) was blended with distilled water in a wort kettle to 7°P original gravity, boiled for 5 min and then cooled down to the pitching temperature of 20°C using a plate cooler. A volume of 50 l wort was pumped into a cylindroconical tank before 1800 ml was filled into each of the 2000 ml bottles for the fermentations. Propagation yeasts were centrifuged (Roto Super 40, Andreas Hettich GmbH & Co. KG, Tuttlingen, Germany) in sterilized 500 ml PPCO centrifuge bottles (Nalgene, Thermo Fisher Scientific, Waltham, MA, USA) at 750 × *g* for 5 min and the supernatant was discarded before pitching. The samples were incubated at 20°C for fermentation. The fermentation time of 144 h was chosen based on previous experiments (Methner et al. 2022). Subsequently, the samples were then transferred to cold storage at 2°C for another 96 h before performing the analytical analyses described in Table 2.

**Table 2.** Analytical methods of the wort and the nonalcoholic beers according to MEBAK^a^.

Analysis	Method	Device
Original gravity, apparent attenuation, and ethanol content	MEBAK WBBM 2.9.6.3	Bending vibration and NIR spectroscopy, Alcolyzer Plus with DMA 5000 X sample 122 (Anton-Paar GmbH, Ostfildern, Germany)
pH	MEBAK WBBM 2.13	pH meter with pH electrode, ProfiLine pH3210 pH meter (Xylem Inc., New York, NY, USA)

a MEBAK® (2012), Editor: Dr F. Jacob: The MEBAK collection of brewing analysis methods: Wort, beer, and beer-based beverages. Collection of methods of the Mitteleuropäischen Brautechnischen Analysenkommission. Self-published by MEBAK.

From the results obtained by the analytical methods shown in Table 2, one-sample *t*-tests were performed using OriginPro 2020 as statistical software to evaluate whether the triplicates of each beer were insignificantly different from each other. If there was no significant difference in the one-sample *t*-test, it may be assumed that a composite sample from the respective triplicates would reveal a statistically significant result in the vitamin B analysis. Therefore, unless significant differences were found, composite samples from the triplicates were used for vitamin B analysis.

The four produced nonalcoholic beers and the corresponding wort were measured for B vitamins, whereas the propagated yeast and the sedimented yeast after fermentation were only quantified in case of the yeast strain S. lud SL17. SGS Institut Fresenius GmbH analyzed the 7°P wort, propagated and sedimented yeast of the yeast strain S. lud SL17 as well as its nonalcoholic beer for all B vitamins except folate using the methods listed in Table 3. Additionally, pantothenic acid, biotin, and cobalamin of the three nonalcoholic beers fermented with the yeast strains C. sat 247, C. sat CSa1, and K. mar 653 were determined by SGS Institut Fresenius GmbH according to the methods in Table 3. Since SGS Institut Fresenius GmbH, Freiburg, Germany is accredited according to DIN EN ISO/IEC 17025, it was scientifically permissible to perform single determinations of the selected B vitamins. Due to the accreditation, the coefficients of variation were calculated from the respective standardized methods and were used for the statistical evaluation.

**Table 3.** Analytical methods of vitamin B used by SGS Institut Fresenius GmbH, Freiburg, Germany, (accreditation according to DIN EN ISO/IEC 17025) for wort, beer, and propagated as well as sedimented yeast.

Analysis	Method—wort/beer	Method—yeast
B_1_–Thiamine	DIN EN 14122, HPLC/Fl	DIN EN 14122, HPLC/Fl
B_2_–Riboflavin	DIN EN 14152, HPLC/Fl	DIN EN 14152, HPLC/Fl
B_3_–Niacin	AOAC 944.13, microbiological	AOAC 944.13, microbiological
B_5_–Pantothenic acid	AOAC 945.74, microbiological	AOAC 945.74, microbiological
B_6_–Pyridoxine	DIN EN 14663, HPLC/Fl	DIN EN 14663, HPLC/Fl
B_7_–Biotin	SOP M 3532, LC-MS/MS	SOP M 655, microbiological, *L. plantarum*
B_12_–Cobalamin	AOAC 952.20/986.23, microbiological	AOAC 952.20/986.23, microbiological

Prior to the quantification of B vitamins for the propagated and the sedimented yeast, the samples were centrifuged for 10 min at 750 × *g* (centrifuge Z 366 K, HERMLE, Wehingen, Germany). The supernatant was discarded and the yeast was resuspended with 20 ml sterile physiological NaCl solution to eliminate the influence of wort or beer. The washing procedure was repeated three times before the analyses were performed.

Folate was analyzed in triplicate in the beer SL17, in the three experimental beers 247, CSa1, and 653, and in the propagated and sedimented yeast of the yeast strain S. lud SL17 as follows. The folate extraction of the samples was performed under dimmed light as described in Striegel et al. (2018). A total of 100–150 mg of the yeast and 500 mg of the liquid samples were used for extraction. For the quantification ^13^C_5_-PteGlu, ^13^C_5_-H_4_folate, ^13^C_5_-5-CH_3_-H_4_folate, ^13^C_5_-5-CHO-H_4_folate, and ^13^C_5_-10-CHO-PteGlu were added in an equal amount of the expected concentration of the unlabeled analytes in the sample. For deconjugation, 900 ml chicken pancreas solution and 400 ml rat serum were added to the sample. The preparation of the solutions for deconjugation was performed according to Striegel et al. (2018). After overnight incubation at 37°C, the samples were boiled in a water bath at 100°C and 10 ml acetonitrile was added once the samples had cooled down. The samples were purified by solid phase extraction (SPE) before being measured by using LC-MS/MS. The LC-MS/MS analysis was carried out on a Shimadzu Nexera X2 UHPLC system (Shimadzu, Kyoto, Japan) with a Raptor ARC-18 column (2.7 µm, 100 × 2.1 mm, Restek, Bad Homburg, Germany) and a Raptor ARC-18 precolumn (2.7 µm, 5 × 2.1 mm, Restek) as a stationary phase. The mobile phase for the binary gradient consisted of (A) distilled water with 0.1% (v/v) formic acid and (B) acetonitrile with 0.1% (v/v) formic acid at a flow rate of 0.4 ml/min. The LC-system was coupled with a triple quadrupole mass spectrometer (LCMS-8050, Shimadzu) and operated in positive ESI mode for all analytes. The specified settings as well as method validation have been described previously (Striegel et al. 2018).

### Determination of BAs

For the analysis of BAs, yeast strains from slant agars were inoculated into 50 ml of sterilized wort made from unhopped malt extract (Weyermann®) at 12.0°P and pH 5.46. Prior to propagation, the wort was sterilized at 100°C for 45 min. After 72 h propagation, the yeast cells were counted using the Cellometer® Vision (Nexcelom Bioscience LLC). For fermentation, each yeast was pitched in triplicate at a rate of 30 × 10^6^ cells/ml in 300 ml of 12.0°P wort from malt extract (Weyermann®) into 500 ml Duran glass bottles (Schott AG). In order to keep the thermal influence on the wort as low as possible for the subsequent measurement of BAs, the malt extract was only diluted with boiling distilled water (100°C) for the fermentation trials while the Duran glass bottles (Schott AG) and glass fermentation airlocks were previously autoclaved at 121°C for 15 min. The fermentations were incubated at 20°C for 8 days before being analyzed for BAs. To ensure that fermentation occurred and for statistical evaluation, the original gravity, apparent attenuation, ethanol content, and pH of the finished beers were determined according to the methods in Table 2. As already described for statistical evaluation of B vitamins, one-sample *t*-tests were performed using OriginPro 2020 as statistical software to evaluate whether there was any significant difference between the triplicates of each beer. If there was no significant difference in the one-sample *t*-test, it may be assumed here too, that a composite sample from the respective triplicates would reveal statistically significant results in the analysis of BAs. Therefore, unless significant differences were found, composite samples from the triplicates were used for the analysis. Moreover, the samples were checked microscopically for microbiological purity. In addition to the 16 investigated yeast strains and the additional reference yeast strain S. cer 68 from Table 1, a wort sample was analyzed as a negative control. The positive control consisted of three pale barley malt kernels, each from three different malt batches added to the wort at the beginning of the fermentation. To determine which microorganisms led to the positive finding of BAs, the beer was fractionally streaked on Wallerstein Nutrient (WLN) agar (Sigma-Aldrich, St. Louis, MO, USA) as well as wort agar and incubated for 48 h at 28°C. Morphologically different individual colonies were identified by a IVD MALDI Biotyper® system solution based on the microflex® LT/SH MALDI-TOF mass spectrometer (Bruker Daltonics GmbH & Co. KG, Bremen, Germany). The corresponding Bruker standard operating procedures were applied for the sample preparations for bacteria and yeasts. The direct transfer method (DT) was used for bacteria and the extended direct transfer method (eDT) for yeasts. SGS Institut Fresenius GmbH, Freiburg, Germany, (accreditation according to DIN EN ISO/IEC 17025) performed the analysis of the BAs histamine, tryptamine, phenylethylamine, isopentylamine, putrescine, cadaverine, tyramine, spermidine, and spermine using HPLC SLMB 1391.1 (Var. 1). The limit of determination was at least 5 mg/l for all BAs analyzed. Since the majority of the measured concentrations were below the limit of determination but above the limit of detection, trends of the individual BAs are presented as result ranges for all 17 of the analyzed beers. The positive control is shown separately.

### Stress tolerance tests

As a preliminary test, the physiological growth of the yeast strains from Table 1 was investigated at 37°C by inoculating each yeast as a pure culture from slant agar into 1 ml of sterile physiological sodium chloride solution and counting them using the Cellometer® Vision (Nexcelom Bioscience LLC). The solutions were adjusted to 1 × 10^6^ cells/ml with sterile physiological sodium chloride solution and fractions streaked in triplicate onto WLN agar (Sigma-Aldrich) plates, which had been incubated for 4 h at 37°C. Subsequently, the agar plates were kept for 7 days at 37°C and were checked for growth at the end of the incubation period. Here, the reference yeast strain S. cer TUM 68 was included as a positive control as the yeast species *S. cerevisiae* as a top-fermenting yeast is known for its ability to grow at 37°C (Lodolo et al. 2008, Hutzler et al. 2015).

The yeast strains that showed growth at the end of the incubation period at a temperature of 37°C were further investigated *in vitro* for human gastric juice tolerance. One yeast strain (S. lud SL17) that did not grow at 37°C was added as a negative control. The selected pure yeast cultures were first propagated for 72 h on a WiseShake orbital shaker (Witeg Labortechnik GmbH) at 80 rpm and 20°C in 50 ml of sterilized 9°P wort prepared from unhopped malt extract (Weyermann®). After propagation, the yeast cells were counted and their viability was determined with the aid of Cellometer ViaStainTM AOPI Staining Solution (Nexcelom Bioscience LLC) using the Cellometer® Vision (Nexcelom Bioscience LLC). The volumes of yeast cell suspensions were calculated and adjusted to 15 × 10^6^ viable cells per ml using fresh, sterile 9°P unhopped wort for the adjustments. A volume of 10 ml of each yeast solution was pipetted into sterile 15 ml Sarstedt tubes (Sarstedt AG & Co. KG, Nümbrecht, Germany). The synthetic gastric juice was prepared according to the instructions of DIN 19738:2000–05 and the formulation is listed in Table 4.

**Table 4.** Composition of synthetic gastric juice according to DIN 19738:2000–05.

Substance	Concentration [mg/100 ml]
KCl	290
NaCl	70
KH_2_PO_4_	27
Pepsin	100
Mucin	300
10% HCl (for pH adjustment)	As required for pH 2

While the inorganic components were prepared as a 10-fold concentrated stock solution and were rediluted with double-distilled water, pepsin from porcine gastric mucosa, lyophilized powder, ≥ 2500 units/mg protein (Sigma-Aldrich), and mucin from porcine stomach (Typ II M1778, Sigma-Aldrich) were stirred in just before adding the yeast sample. The experiments were carried out in duplicate.

Each 100 ml of synthetic gastric juice was temperature-controlled at 37°C in sterile 300 ml wide-neck flasks in an automatically temperature-controlled water bath (heating bath B-490, Büchi Labortechnik AG, Flawil, Switzerland). Once the gastric juice reached the target temperature, 10 ml yeast suspension was added by stirring and the pH was adjusted to 2.0 with 10% hydrochloric acid. The suspension was kept at 37°C in a shaking water bath (Gesellschaft für Labortechnik mbH, Burgwedel, Germany) with an integrated thermostat at a frequency of 200/min for 10 min before being adjusted to a pH of 7.5 with solid sodium bicarbonate. Fine adjustment was performed with saturated NaHCO_3_ solution. The solution was shaken until the pH value was stable at 7.5 before 10 ml of the solution was transferred to a sterile 15 ml Sarstedt tube (Sarstedt AG & Co. KG). Excess sample material was discarded. A volume of 10 ml of each sample material was held at 37°C for 2 h before being centrifuged at 750 × *g* (centrifuge Z366K, Hermle Labortechnik, Wehingen, Germany) for 5 min, washed with sterile physiological sodium chloride solution, and resuspended to 10 ml total volume. A small amount was fractionally streaked on YM agar [0.3% malt extract (Merck, Darmstadt, Germany), 0.3% yeast extract (Sigma-Aldrich), 0.5% peptone from casein, pancreatic digest (Sigma-Aldrich), 1.0% ᴅ(+)-glucose anhydrous (Carl Roth, Karlsruhe, Germany), 2.0% agar (sifin diagnostics gmbh, Berlin, Germany), 95.9% double-distilled water], preincubated for 4 h at 37°C with an inoculation loop under sterile conditions and incubated at 37°C for 7 days. If yeast colonies grew on the YM agar, they were examined microscopically under a Zeiss bright field microscope. Moreover, the viability of the yeasts was investigated as a percentage right after the 2 h incubation at 37°C and subsequent washing procedure. For this purpose, the Cellometer® Vision (Nexcelom Bioscience LLC) was initially used and sample preparation using Cellometer ViaStain^TM^ AOPI Staining Solution (Nexcelom Bioscience LLC) according to the manufacturer’s instructions. This method was supplemented by counting the cells with a Zeiss brightfield microscope in a hemocytometer (Thoma, depth of 0.100 mm) by diluting the samples 1:1 with methylene blue solution beforehand (Herrmann and Schindler 2022).

## Results and discussion

### Vitamin B concentrations in selected nonalcoholic beers and yeast strain S. lud SL17

Initially, the one-sample *t*-test was performed as described in the materials and methods section. Without exception, the mean population of each triplicate of the beers and the corresponding wort was not significantly different from the test mean at the 0.05 level (cf. Table S1, Supporting Information). Consequently, composite samples could be produced from the respective triplicates to gain statistically significant results.

**Figure 1.**
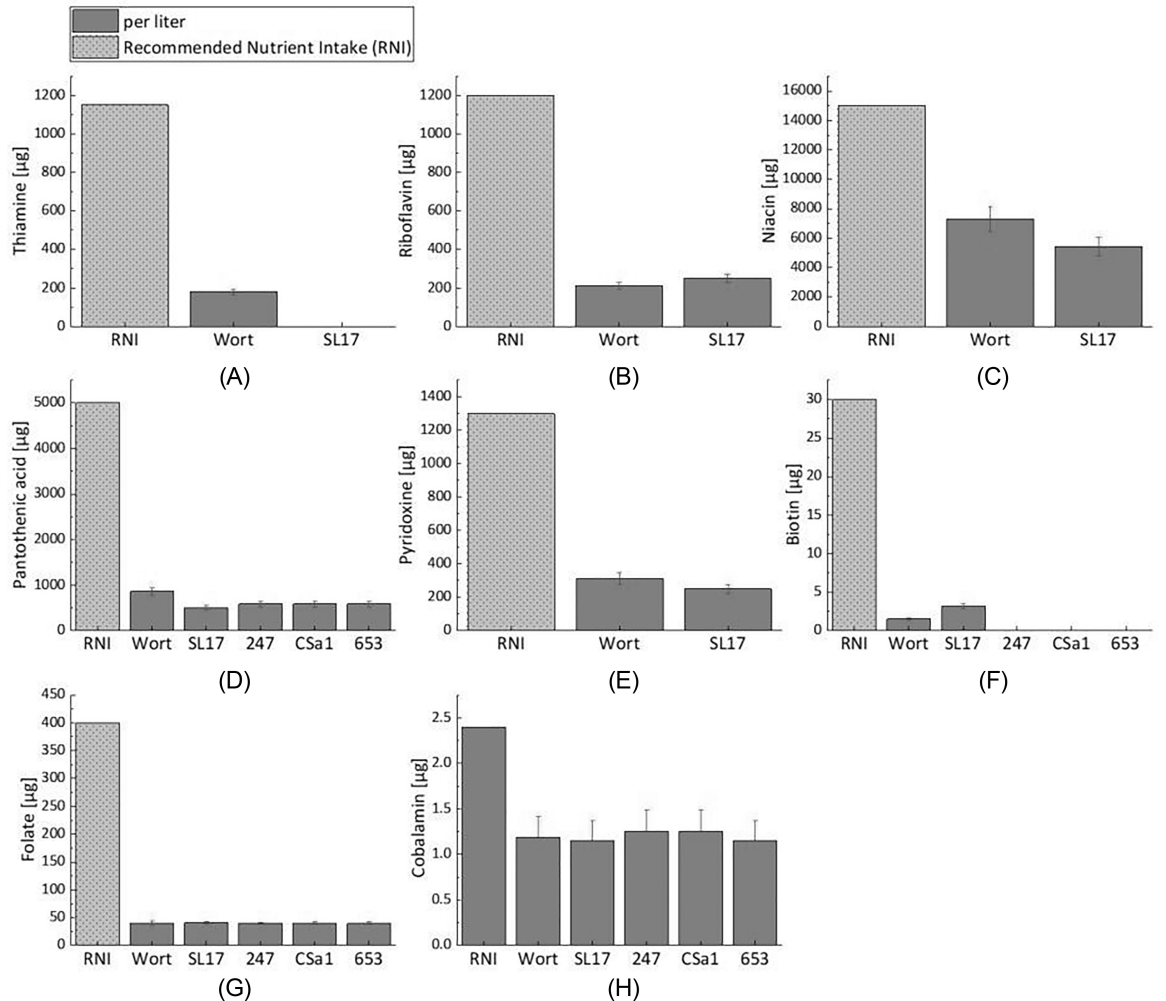
Concentrations in µg/l of the B vitamins thiamine (A), riboflavin (B), niacin (C), pantothenic acid (D), pyridoxine (E), biotin (F), folate (G), and cobalamin (H) analyzed in wort as well as in nonalcoholic beers fermented with the yeast strains *S. ludwigii* SL17, *C. saturnus* 247, *C. saturnus* CSa1, and *K. marxianus* 653. Thiamine, riboflavin, niacin, and pyridoxine analyses were only conducted for wort and nonalcoholic beers fermented with the yeast strain *S. ludwigii* SL17. RNI according to FAO and WHO (Food and Agriculture Organization (FAO)/World Health Organization (WHO) Nutrition Division 2001). Enlarged representations of the individual diagrams can be found in Figure S1 (Supporting Information).

In Fig. 1, the concentrations of the eight B vitamins in the wort as well as in the corresponding beers are depicted. Concentrations of B vitamins are shown in µg/l and are compared to the Recommended Nutrient Intake (RNI) published by the Food and Agriculture Organization (FAO) and the World Health Organization (WHO; Food and Agriculture Organization (FAO)/World Health Organization (WHO) Nutrition Division 2001). The RNI only serves as an indicator as different studies make different recommendations in some cases. For example, a study from 2019 raised recommended levels of vitamin B12 intake for adults to 4.0 µg/day (Ströhle et al. 2019). Moreover, the RNI indicated applies to adults over the age of 18. In case there are different values for women and men, the mean value is shown. Raw data can be found in Table S2 (Supporting Information). The selected focus on the B vitamins pantothenic acid, biotin, folate, and cobalamin for the four nonalcoholic beers was due to the fact that biotin, folate, and cobalamin play a significant nutritional role in human metabolism, which will be explained in more detail. Pantothenic acid was additionally determined in all beers, as this vitamin is known to be essential for yeast growth of the species *S. cerevisiae* (Williams et al. 1940). All other B vitamins were analyzed only for the beer SL17, since the yeast strain S. lud SL17 is already commercially used in practice to produce nonalcoholic beers.

The diagrams in Fig. 1 generally show that none of the beers exceeded the RNI according to FAO/WHO. However, it is noticeable that the biotin concentration in beer SL17 was approximately twice as high as in the initial wort, which was not the case for any other B vitamin and is depicted in diagram F. While only 1.53 µg/l (CV ± 0.156) biotin was measured in the wort used for fermentation, the beer SL17 contained 3.17 µg/l (CV ± 0.323) biotin. Bamforth (2002), who reviewed the vitamin B concentration ranges in beer, found a common range from 2 to 15 µg/l. Accordingly, the biotin concentration is within the expected range. The RNI is 30 µg/l, and so approximately 10% of the daily requirement would be covered by consuming 1 l of the nonalcoholic beer. Based on the low biotin concentration in the initial wort, it could be assumed that the yeast strain *S. ludwigii* SL17 had the ability to synthesize and release biotin or that biotin reserves stored in the yeast cell were released during fermentation. It is noticeable that the two *C. saturnus* yeast strains 247 and CSa1 as well as the *K. marxianus* yeast strain 653 were unable to release biotin. They might have utilized it for their own metabolic process since no more biotin could be detected in these three beers. The fact that biotin is present in the raw materials of brewer’s wort and is taken up by the yeasts as an important growth factor was already described by Lynes in 1948 (Lynes and Norms 1948). Still, Wu et al.(2005) assumed that some yeasts might be able to synthesize biotin themselves. In their study, sake yeasts were able to produce biotin so they speculated that specific genes needed to be present to allow biotin synthesis. Hall and Dietrich (2007) described that the functional gene cluster *BIO1*–*BIO6* is required for yeasts to be biotin-prototrophic and to produce the vitamin accordingly. Many *S. cerevisiae* yeast strains can partially synthesize biotin, but *de novo* synthesis is not possible. Partial synthesis requires certain precursors to be present in the culture medium from which biotin can be formed (Stolz 2009). It cannot be completely excluded that S. lud SL17 could use these precursors from the wort to synthesize biotin or that the yeast strain carries the specific gene cluster. Still, this would have to be investigated in future research. Nevertheless, in the further course of the study, in addition to the beer, the yeast strain S. lud SL17 was also examined for biotin in order to be able to draw further conclusions.

Concentrations of around 40 µg/l folate were quantified in both the wort and the four beers (cf. Fig. 1, diagram G). Therefore, the yeasts do not appear to utilize significant amounts of folate during their fermentation metabolism nor do they seem to synthesize this vitamin. Thus, the folate most likely originated from the raw material malt and was responsible for the concentration found in the beer. A study by Koren et al. (2021) revealed that barley is a natural source of folate and, depending on the quality of the malt and the malting process, barley malt is also an adequate source of this vitamin. Moreover, Narziss et al. (2017) stated that 70–100 µg/l folate in beer can originate from the raw material malt. Based on the results, only around 10% of the RNI for folate would be achieved by consuming 1 l of any of the four investigated nonalcoholic beers. Folate could still supplement a balanced diet and it can be generally concluded that the nonalcoholic beers represent a valuable source of this B vitamin. A common folate range in European beers is between 30 and 64 µg/l, which is consistent with the measured values in this study (Owens et al. 2007). The range can be wide since, in addition to folate from the raw material malt, some yeast species like the traditional brewing yeast *S. cerevisiae* are known to synthesize folate (Jacob et al. 2019). Both folate and biotin are essential B vitamins involved in catalytic properties in the human metabolism due to their function as obligatory cofactors of enzymes (Baumgartner 2013). A folate deficiency can, e.g. lead to cardiovascular diseases, cancer or cognitive dysfunctions (Ebara 2017), whereas a biotin deficiency can lead to negative effects on the immune system, cell proliferation and lipid metabolism among other things (Zempleni et al. 2009). Moreover, biotin most likely plays a role in regulating gene expression. Deficiency may also be associated with neurological diseases (León-Del-Río 2019).

Based on the results of Fig. 1, the B vitamins thiamine, riboflavin, niacin, pantothenic acid, pyridoxine, and cobalamin are derived from the raw material barley malt, since none of these B vitamins in the nonalcoholic beers were notably above the concentration in the initial brewer’s wort. While diagram A shows that thiamine was completely utilized by the yeast strain S. lud SL17 for its own metabolism, riboflavin stayed unaltered within the scope of the measurement variations (Fig. 1, diagram B) and on average around 20% of pyridoxine (Fig. 1, diagram E) was taken up by the yeast strain. For niacin (Fig. 1, diagram C), the yeast strain S. lud SL17 required on average about 25% of this vitamin present in the wort for its own metabolic processes. Pantothenic acid (Fig. 1, diagram D) was absorbed to a small extent from the wort by all yeast strains studied during fermentation. Although this B vitamin is essential for yeast growth, the pantothenic acid present in the wort was sufficient to meet the demand of the investigated yeasts. Between 10% and 35% of the RNI of riboflavin, niacin, pantothenic acid, and pyridoxine could be quantified in the beers per liter. Although these amounts were not sufficient to meet the RNI, the beers could still serve as a supplement to meet the daily vitamin B requirements. The same could apply to cobalamin (diagram H), where similar concentrations were measured in both the wort and the beers. Cobalamin appeared to remain unaffected in the wort during fermentation by the four yeast strains within the limits of measurement inaccuracies. Consumption of 1 l of one of the four nonalcoholic beers per day could cover around 50% of the RNI according to the FAO/WHO. However, it must be considered that, e.g. with regard to cobalamin, there are more recent studies that recommend even 4 µg per day (Ströhle et al. 2019). It is known that beer naturally contains cobalamin in a concentration range between 3 and 30 µg/l (Bamforth 2002, Heimpel 2003). Most probably it originates from the raw material barley malt as besides the chemical production pathway, only prokaryotes are able to synthesize cobalamin (Friedmann and Cagen 1970, Eschenmoser 1974, Lawrence and Roth 1996). Plants do not seem to be able to produce cobalamin (Duda et al. 1967). Therefore, microbiological contamination on the plants is most likely responsible for the formation of this vitamin (Watanabe et al. 1991). In a work by Krahl (2010), it was revealed that microorganisms grew to varying degrees during germination on the grains. This caused the differences in the vitamin B12 concentration in the beers as the quantity of malt was the decisive influencing factor. This could explain why only approximately 1.2 µg/l cobalamin was measured in the wort as well as in the nonalcoholic beers as the original gravity was adjusted to 7°P. The quantity of barley malt used was therefore significantly lower than in a regular beer.

**Figure 2.**
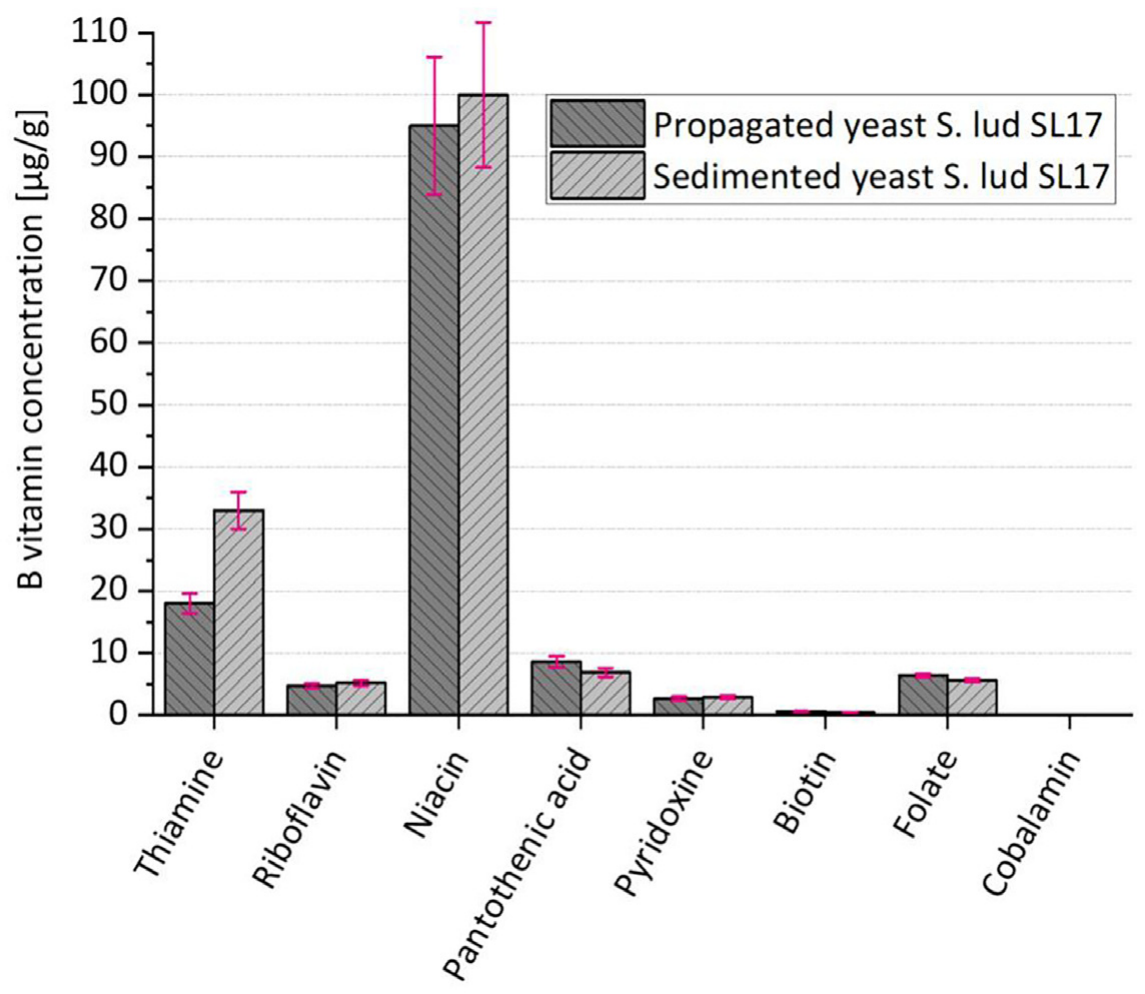
Concentrations in µg/g of the B vitamins thiamine, riboflavin, niacin, pantothenic acid, pyridoxine, biotin, folate, and cobalamin analyzed in the propagated yeast *S. ludwigii* S. lud SL17 as well as the sedimented yeast S. lud SL17.

It was not clarified in the previous investigations whether the yeasts store the B vitamins absorbed from the wort or immediately consumed them during their own yeast cell metabolism. Based on the complete B vitamin analysis of the yeast strain S. lud SL17, both the propagated yeast before fermentation and the sedimented yeast after fermentation were analyzed for all eight B vitamins. The results are depicted in Fig. 2. Raw data can be found in Table S3 (Supporting Information).

Thiamine, which was completely taken up from the wort by the yeast strain S. lud SL17 during fermentation (cf. Fig. 1, diagram A), was either adsorbed or absorbed by the yeast. Although on average 18 µg/g thiamine was already quantified in the propagated yeast, a value almost twice as high (around 33 µg/g) was present in the sedimented yeast after fermentation. Therefore, approximately 15 µg/g thiamine accumulated in the yeast. The yeast mass after fermentation was 16.4 g on average (± 0.4). Accordingly, approximately 240 µg/l thiamine was absorbed from the wort. Since a total of around 330 µg thiamine was originally present in the 1.8 l wort sample, it could be assumed that part of the vitamin B_1_ from the wort was already utilized for yeast cell metabolism, while the rest was stored inside the cells. The yeast cell, therefore, appeared to have a storage function for this B vitamin. Riboflavin, niacin, pyridoxine, and cobalamin remained unaltered in the yeast by considering the coefficients of variation. However, since Fig. 1 showed that niacin and pyridoxine were partially reduced during fermentation, these two B vitamins appeared to have been utilized immediately. For riboflavin and cobalamin, it could be assumed that S. lud SL17 did not require these B vitamins for its metabolism. Since no cobalamin could be measured in either the propagated or the sedimented yeast, it was possible to confirm the assumption that the cobalamin quantified in the beers originated from the wort, which fits the findings from existing literature (Krahl 2010). Pantothenic acid was reduced by about 20% during fermentation (cf. Fig. 1). Therefore, it could be deduced that S. lud SL17 immediately metabolized the pantothenic acid taken up from the wort and, in addition, used its own reserves. The folate concentration in the sedimented yeast decreased by approximately 10%, showing a slight consumption by the yeast during fermentation. Biotin decreased by 30% during fermentation in the yeast strain S. lud SL17. At a yeast mass of 16.4 g on average, around 3.1 µg biotin was released into the wort or utilized by the yeast for its own metabolism. Since there was already approximately 2.8 µg of biotin in 1.8 l of wort, it could be assumed that the yeast released a small amount of biotin during fermentation and needed the larger share for its own cell metabolism. Based on this additional investigation, it is to be expected that the yeast strain S. lud SL17 did not possess the ability to synthesize biotin itself and it had a storage function inside the cell.

### Concentrations of BAs in selected nonalcoholic beers

Prior to the measurement of BAs, to ensure that fermentations with the 17 yeast strains from Table 1 proceeded, the beers were analyzed for original gravity, ethanol, apparent attenuation, and pH after fermentation. The individual results can be found in Table S4 (Supporting Information). In summary, the fermentations proceeded as expected. The four maltose- and sucrose-negative yeast strains C. mis 238, C. mis CM1, K. ser 3C4, and P. klu PK1 showed the lowest apparent attenuation as well as the lowest ethanol concentrations. As a maltose-positive yeast, the reference yeast strain S. cer 68 exhibited by far the highest apparent attenuation and ethanol concentration, whereas the maltose-negative yeasts showed values in between. The original gravity was deliberately selected at approximately 12°P so that the ethanol content of all beers exceeded 0.50% (v/v; cf. Table S4, Supporting Information). In the event that BAs were detected, it was necessary to ensure that the limit value of 50 mg/kg total BAs (Loret et al. 2005) in the beers was not exceeded within the nonalcoholic range ≤ 0.50% (v/v).

**Figure 3.**
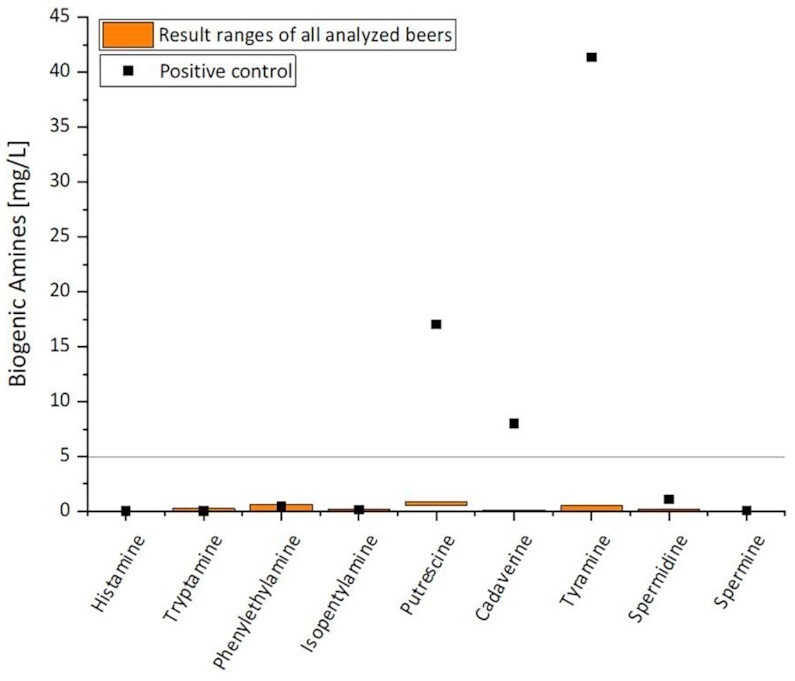
BAs histamine, tryptamine, phenylethylamine, isopentylamine, putrescine, cadaverine, tyramine, spermidine, and spermine in mg/l of 17 different yeast strains (cf. Table 1) presented as result ranges due to concentrations below the limit of determination (limit of determination ≥ 5.0 mg/l) as well as a positive control shown individually.

Regarding the one-sample *t*-tests, the *t*-tests were also initially performed for BAs as described in the “Materials and methods” section. Since, without exception, the mean population of each triplicate of the beers did not differ significantly at the 0.05 level from the test mean (cf. Table S5, Supporting Information), composite samples from the respective triplicates were permitted in order to gain statistically significant results with regard to the selected BAs. As mentioned in the “Materials and methods” section, the majority of the quantified results of the BAs were below the limit of determination but above the limit of detection. The limit of determination is set by SGS Institut Fresenius GmbH at ≥ 5 mg/l. Without exception, all beers produced with the 17 experimental yeast strains from Table 1 were below the limit of determination and only the positive control exhibited concentrations above this threshold. Therefore, only the result ranges of the 17 yeast strains are shown as a summary in Fig. 3, whereas the positive control is shown individually.

While histamine and spermine were below the limit of detection for all 17 yeast strains without exception, the other seven BAs were formed in very small amounts or were already present in the wort sample. Minor amounts of putrescine, cadaverin, tyramine, and spermidine were, therefore, quantified in the brewer’s wort. Nevertheless, the concentrations of the analyzed nine BAs were, without exception, below the limit of determination (5.0 mg/l) in the wort. It is known from previous studies that besides microbial formation during fermentation, BAs can also be formed during malting (Gasarasi et al. 2003). While putrescine and cadaverine remained almost unaltered in the beer samples, some of the yeast strains seemed to be able to reduce tyramine and spermidine either partially or even completely during fermentation as is already discussed in the literature (Benito et al. 2015, Ivit et al. 2018). Isopentylamine was formed in small amounts by almost all the yeast strains examined, while only two yeast strains could synthesize marginal amounts of tryptamine and phenylethylamine. These results give a first indication that the investigated yeast strains, without exception, could not build up notable amounts of BAs from wort precursors during fermentation. In total, the highest concentration of BAs in the beers produced was below 3 mg/l. This is significantly lower than the average concentration of regular uncontaminated beers, which are reported in literature to be between 8 and 30 mg/l (Narziss et al. 2017). In beers with 12°P original gravity, the histamine content is usually 0.15–0.20 mg/l, while the histamine concentration for the investigated beers was so low that it could not be detected. Accordingly, no adverse health effects are suspected from BAs in the nonalcoholic beers produced with the non-*Saccharomyces* yeasts investigated. In addition, the BA results of the beer fermented with the *Saccharomyces* yeast strain S. cer 68 showed no significant differences from the non-*Saccharomyces* yeasts.

The positive control, which was composed of three pale barley malt kernels each from three different batches added to the brewer’s wort at the beginning of the fermentation, demonstrated that the three BAs cadaverine, putrescine, and tyramine could be detected at concentrations significantly above the limit of determination of 5 mg/l. In Fig. 3, cadaverine is just under 8 mg/l, while a concentration of 17 mg/l was measured for putrescine and about 41 mg/l for tyramine. The exact concentrations can be viewed in Table S6 (Supporting Information). For all other BAs of the positive control, the concentrations were below the limit of determination. Using MALDI-TOF, it was found that the wort contained microorganisms including *Lactobacillus plantarum, Candida krusei*, and *Acetobacter* sp., which originated from the pale malt grains. BAs are mainly formed by microbial decarboxylation of amino acids and the formation is often observed from lactic acid bacteria (Brink et al. 1990, Alcaide-Hidalgo et al. 2007, Spano et al. 2010, Sumby et al. 2014). *Lactobacillus plantarum* is known from the literature for its ability to produce putrescine from the amino acid arginine which is found in brewer’s wort (Arena and Manca de Nadra 2001, Ferreira and Guido 2018). Besides putrescine, cadaverine, and tyramine can be formed by *L. plantarum* (Bonnin-Jusserand et al. 2012, Alan et al. 2018). The BAs tyramine and cadaverine are formed from the amino acids tyrosine and lysine, which are also present in brewer’s wort (Ferreira and Guido 2018, Vicente et al. 2021). *Candida krusei* (*Pichia kudriavzevii*) possess decarboxylase activity against the amino acids tyrosine and lysine (Delgado-Ospina et al. 2020) so it could be speculated that this yeast species might have been responsible for the elevated tyramine and cadaverine concentrations in the positive control. However, further research would need to be conducted in the future to prove this speculation. There is no literature to date about the formation of BAs by acetic acid bacteria such as *Acetobacter* sp. On the contrary, studies may indicate that BAs could be degraded during acetic fermentation (Landete et al. 2007, Ordóñez et al. 2013, 2015).

### Stress tolerance tests

Before the 17 yeast strains listed in Table 1 were investigated *in vitro* for their tolerance to synthetic human gastric juice, a pretest was performed to first elucidate the ability of the individual yeast strains to grow at 37°C, based on human body temperature. Table 5 lists the results after 7 days of incubation.

**Table 5.** Growth of the investigated yeast strains at 37°C on WLN agar after 7 days of incubation.

Yeast strain abbreviation	Growth at 37°C
C. fab 5640	++
C. fab 5650	++
C. mis 238	−
C. mis CM1	−
C. sat 247	−
C. sat 4549	−
C. sat CSa1	−
K. ser 3C4	−
K. lac G9K	−
K. mar 653	++
L. klu 3082	++
P. klu PK1	++
S. cer 68	++
S. lud SL17	−
S. fib Lu27	++
S. pom G10S	++
T. del 1I6	−

(++) growth observed, (-) no growth.

**Figure 4.**
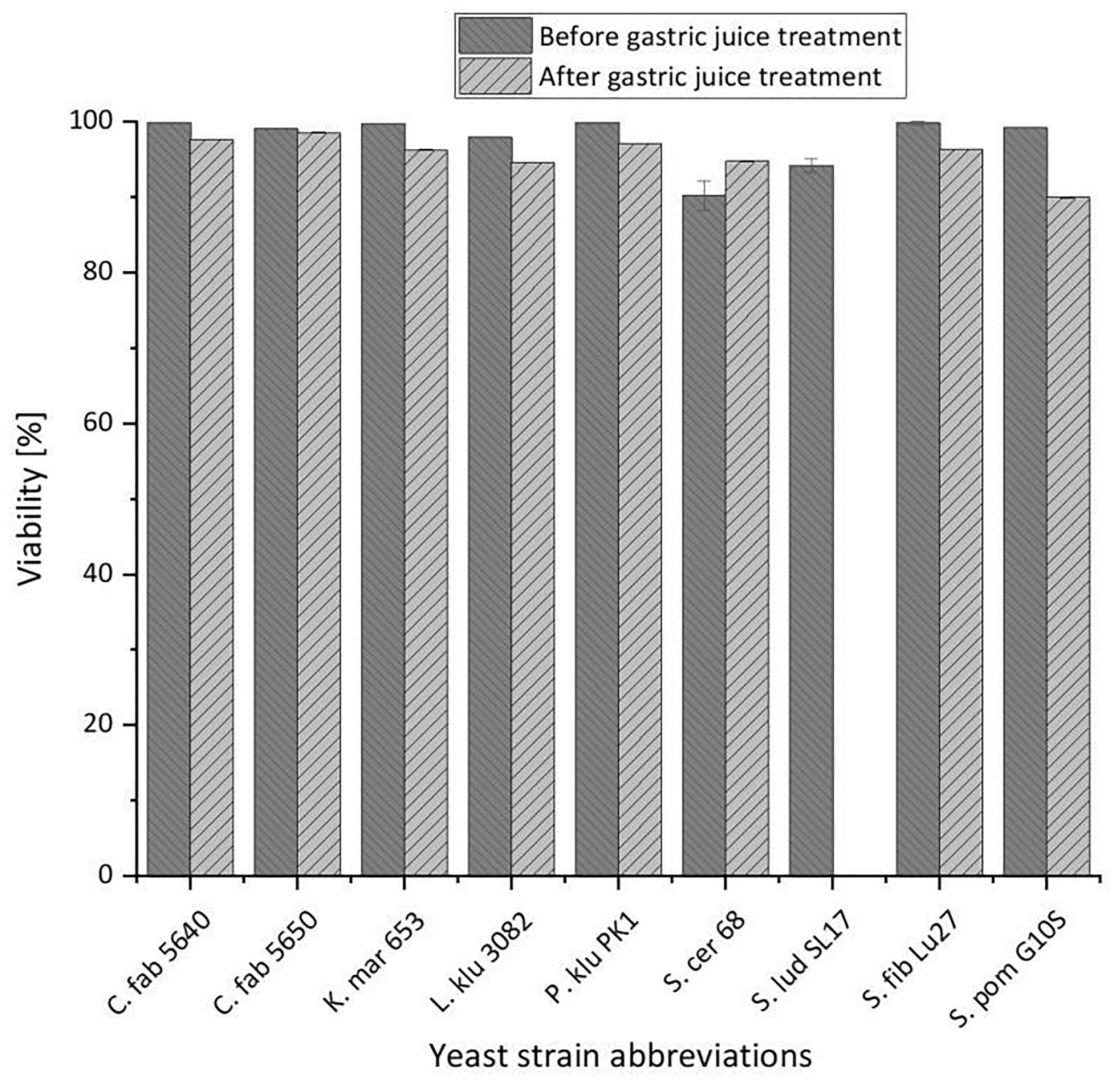
Viability (%) by counting cells with a Zeiss bright-field microscope in a Thoma counting chamber with methylene blue before and after synthetic gastric juice treatment of the selected yeast strains C. fab 5640, C. fab. 5650, K. mar 653, L. klu 3082, P. klu PK1, S. fib Lu27, S. pom G10S, and the positive control S. cer 68 as well as negative control S. lud SL17.

A total of eight of the total 17 yeast strains from Table 5 showed growth on WLN agar within 7 days at an incubation temperature of 37°C. While S. cer 68 used as a positive control showed growth, the two strains of the yeast species *C. fabianii*, K. mar 653, L. klu 3082, P. klu PK1, S. fib Lu27, and S. pom G10S were also able to proliferate at the selected incubation temperature. In case yeasts are not viable at 37°C, potential pathogenicity to the human organism is largely excluded. Therefore, only the previously mentioned eight yeast strains were considered for the following experiment in the synthetic gastric juice environment. The yeast strain S. lud SL17 was included as a negative control. Figure 4 shows whether the selected nine yeast strains survived the synthetic gastric juice treatment at 37°C as described in the methods section. The viabilities of the yeast cells were analyzed before and 2 h after the treatment to study the resistance of the yeast strains to the synthetic gastric juice and to draw a direct comparison.

Figure 4 reveals that the viability of all yeast strains examined before synthetic gastric juice treatment was > 90%. The yeast strains C. fab 5640, C. fab. 5650, K. mar 653, P. klu PK1, S. fib Lu27, and S. pom G10S even showed viabilities > 99%. The viability of the yeast strain L. klu 3082 was close to 98%, while the two reference yeast strains S. lud SL17 and S. cer 68 were between 90% and 95% viability (± 2.0%). Besides the methylene blue method, viability was also determined by the cell counting method described in the methods chapter using the AOPI staining solution containing propidium iodide. Both methods gave similar results before the synthetic gastric juice treatment. However, following synthetic gastric juice treatment for 10 min at 37°C, components from the synthetic gastric juice fluoresced as well as the living yeast cells, giving a consistent viability of 100%. Consequently, only the method using methylene blue was suitable to measure accurate viability in this case.

Looking at the viability decreases in Fig. 4, in addition to the decrease in viability for the negative control, only the yeast strain S. pom G10S showed a decrease in viability of just over 10%. For the other investigated yeast strains, the viability decreased by only 0.5%–3.6%, showing that the influence of the synthetic gastric juice treatment had a minor effect on the viability of the yeast cells. The positive control S. cer 68 increased in viability by almost 5%. Therefore, the yeast cells were presumably able to proliferate after the 10-min synthetic gastric juice treatment and the 2-h regeneration at a pH of 7.5 and a temperature of 37°C. This was likely because 10 ml of the yeast cell suspension in the propagation wort was added to the synthetic gastric juice, thus still providing nutrient substrate for the yeasts to grow on. The fact that the negative control S. lud SL17 showed no viability at all after the treatment with synthetic gastric juice could be attributed to a higher sensitivity to the low pH of 2,which may have led to a denaturation of functional proteins in the cell membrane, causing the yeast cells to lose functionality (Vilgis 2020).

The retention time of the yeast suspensions in the synthetic gastric juice was deliberately limited to 10 min, since previous studies revealed that liquids have a short retention time in the stomach. For example, Best and Conheim described in 1910 that liquids leave the stomach rapidly in both empty and filled states (Best and Cohnheim. 1910). This statement was later substantiated by Minami and Maccallum. They found that liquids leave the empty stomach according to first-order kinetics with a half-life of about 10–20 min (Minami and Mccallum 1984), whereby no gender-specific difference could be observed (Bennink et al. 1998). Nevertheless, it was found that the speed of gastric emptying depends on its filling state with food pulp as an empty stomach behaves differently to a stomach filled with food pulp (Minami and Mccallum 1984). For this study, an empty stomach with the shortest half-life of 10 min was assumed as in case any of the investigated yeast strains could be harmful for human health, a short gastric emptying speed should be considered.

In addition to investigating the short-term response to the gastric environment, the investigation examined whether the selected yeast strains were able to proliferate after the synthetic gastric juice treatment. For this purpose, the yeast suspensions were streaked on YM agar as described in the methods chapter. Table 6 presents the results.

**Table 6.** Growth at 37°C on YM agar of the nine selected yeast strains (including S. cer 68 as positive and S. lud SL17 as negative control) after synthetic gastric juice treatment and additional incubation for 7 days.

Yeast strain abbreviation	Growth at 37°C after gastric juice treatment
C. fab 5640	++
C. fab 5650	++
K. mar 653	++
L. klu 3082	++
P. klu PK1	++
S. cer 68	++
S. lud SL17	−
S. fib Lu27	++
S. pom G10S	++

(+) growth observed, (-) no growth.

The results reveal that, apart from the negative control S. lud SL17, the other eight yeast strains were able to continue growing at 37°C after synthetic gastric juice treatment. Microbiological examination using brightfield microscopy clearly identified the yeast cells, which showed that the formed colonies were not caused by bacterial contamination. Consequently, the yeast strains C. fab 5640, C. fab 5650, K. mar 653, L. klu 3082, P. klu PK1, S. fib Lu27, and S. pom G10S were most likely to survive in the gastric environment and subsequently proliferated at 37°C. Certainly, further studies would need to be performed to confirm this statement. However, the results provide an initial indication of this potential. Additionally, further experiments would also be needed to establish whether the yeast strains could also survive and proliferate in the human intestine. Since seven out of the 16 investigated non-*Saccharomyces* yeasts showed high viability at 37°C and after a 10-min *in vitro* treatment in synthetic gastric juice, further investigations into safety considerations of the yeasts should be conducted. These would include, e.g. studies of toxin expression and virulence genes. With regard to the two yeast strains K. mar 653 and S. pom G10S, it could be assumed that they do not cause any harm to the human organism, as they have QPS status according to EFSA. They could even potentially serve as probiotic microbes. However, extensive investigations would be necessary to evaluate this. In 2006, the FAO published a comprehensive systematic guideline for the evaluation of probiotic microorganisms (Food and Agriculture Organization of the United Nations (FAO)—Nutrition Division 2006). Staniszewski and Kordowska-Wiater (2021) summarized the characteristics that probiotic strains need to have in order to be labeled as such. Non-*Saccharomyces* yeast species such as *K. marxianus* (Fonseca et al. 2008, Hatoum et al. 2012, Cissé et al. 2019, Motey et al. 2020), *K. lactis* (Fadda et al. 2017, Oliveira et al. 2019), *K. servazzii* (Ben Taheur et al. 2021), *S. fibuligera* (Lakshmi Ragavan and Das 2017), *S. pombe* (Gil-Rodríguez et al. 2015), and *T. delbrueckii* (Pereira Andrade et al. 2019, Agarbati et al. 2020) appear increasingly in the context of probiotic or potentially probiotic yeasts. There are currently no study results on this subject relating to *L. kluyveri* and *C. fabianii*. Furthermore, it must be kept in mind that probiotic properties are strain specific (Agarbati et al. 2020).

## Conclusion

In this study, the metabolic properties of selected non-*Saccharomyces* yeasts suitable for producing nonalcoholic beers were investigated for B vitamins, BAs and tolerance to synthetic human gastric juice *in vitro*. With the exception of thiamine, all other analyzed B vitamins, which derived most likely from the raw material malt, were detected in the beer fermented with the yeast strain *S. ludwigii* TUM SL17, covering at least 10% of the RNI per liter. Cobalamin, folate, and pantothenic acid were also measured in nutritionally relevant amounts in the nonalcoholic beers 247, CSa1, and 653 in concentrations comparable to those in the beer SL17. Therefore, B vitamins in nonalcoholic beers fermented with non-*Saccharomyce*s yeasts could serve as a supplement for a balanced diet as they represent a valuable source of B vitamins. The selected BAs stayed far below the limit of determination of 5 mg/l in all investigated beers without exception, while histamine was even below the limit of detection and could not be detected. Consequently, the yeast strains investigated are not capable of synthesizing relevant amounts of the nine analyzed BAs during fermentation, which means that no negative health impact of the beers can be traced back to these BAs. Furthermore, out of the 16 experimental yeasts, seven strains, namely *C. fabianii* 5640, *C. fabianii* 5650, *K. marxianus* 653, *L. kluyveri* 3082, *P. kluyveri* PK1, *S. fibuligera* Lu27, and *S. pombe* G10S, revealed they were capable of proliferating at 37°C and tolerating the conditions of synthetic human gastric juice during *in vitro* experiments. As a precaution, and to eliminate any potential safety concerns for consumers, these yeast strains should be investigated further regarding their use to produce nonalcoholic beers.

## Supplementary Material

foac042_Supplemental_File
