# Supplementary material for: Investigations into metabolic properties and selected nutritional metabolic byproducts of different non-Saccharomyces yeast strains when producing nonalcoholic beer
Source: FEMS Yeast Res. 2022 Aug 25;22(1):foac042. doi: 10.1093/femsyr/foac042 (PMC9629496; doi:10.1093/femsyr/foac042)
Supplement: foac042_Supplemental_File [file foac042_supplemental_file.pdf]

## Supplementary Material

Table S1. One-sample *t*-test results with Prob > |t| of original wort [°P], ethanol % [(*v*/*v*)], apparent attenuation [%] and pH values for subsequent vitamin B analysis of beers fermented with four different non-*Saccharomyces* yeast strains *S. ludwigii* S. lud SL17, *C. saturnus* C. sat 247, *C. saturnus* C. sat CSa1 and *K. marxianus* K. mar 653.

| Analyzed Value | Prob > \|t\| | | | |
|---|---|---|---|---|
| | S. lud SL17 | C. sat 247 | C. sat CSa1 | K. mar 653 |
| Original Wort [°P] | 2.37E-05 | 1.58E-06 | 8.98E-07 | 2.35E-07 |
| Ethanol % [(*v*/*v*)] | 4.50E-05 | 0 | 5.33E-05 | 8.26E-05 |
| Apparent Attenuation [%] | 2.26E-05 | 0 | 6.82E-06 | 7.01E-05 |
| pH Value | 2.80E-05 | 4.41E-06 | 0 | 5.26E-07 |

At the level < 0.05 the population mean (n = 3) does not differ significantly from the test mean (0).

Table S2. Analyzed concentrations [µg/L] of the B vitamins thiamine ($B_1$), riboflavin ($B_2$), niacin ($B_3$), pantothenic acid ($B_5$), pyridoxine ($B_6$), biotin ($B_7$), folate ($B_9$) and cobalamin ($B_{12}$) analyzed in wort as well as in non-alcoholic beers fermented with the yeast strains *S. ludwigii* SL17, *C. saturnus* 247, *C. saturnus* CSa1 and *K. marxianus* 653. Thiamine, riboflavin, niacin and pyridoxine analyses were only conducted for wort and non-alcoholic beer fermented with the yeast strain S. ludwigii SL17. Recommended Nutrient Intake (RNI) according to the Food and Agriculture Organization (FAO) and the World Health Organization (WHO), 2001. SGS Institut Fresenius GmbH, Freiburg, Germany who performed the analyses is accredited according to DIN EN ISO/IEC 17025. Due to the accreditation, the coefficients of variation (CV) were calculated from the respective standardized methods and were used for the statistical evaluation.

| B Vitamin [µg/L] | RNI | Wort | CV Wort | S. lud SL17 | CV S. lud SL17 | C. sat 247 | CV C. sat 247 | C. sat CSa1 | CV C. sat CSa1 | K. mar 653 | CV K. mar 653 |
|---|---|---|---|---|---|---|---|---|---|---|---|
| $B_1$ | 1150 | 180 | 16.2 | < 100 | 0 | n.a. | n.a. | n.a. | n.a. | n.a. | n.a. |
| $B_2$ | 1200 | 210 | 18.27 | 250 | 21.75 | n.a. | n.a. | n.a. | n.a. | n.a. | n.a. |
| $B_3$ | 15000 | 7280 | 851.8 | 5430 | 635.3 | n.a. | n.a. | n.a. | n.a. | n.a. | n.a. |
| $B_5$ | 5000 | 860 | 87.7 | 500 | 51 | 580 | 59.2 | 580 | 59.2 | 580 | 59.2 |
| $B_6$ | 1300 | 310 | 33.2 | 250 | 26.8 | n.a. | n.a. | n.a. | n.a. | n.a. | n.a. |
| $B_7$ | 30 | 1.53 | 0.156 | 3.17 | 0.323 | < 1.0 | 0 | < 1.0 | 0 | < 1.0 | 0 |
| $B_9$ | 400 | 39.6 | 4.00 | 40.8 | 2.40 | 39.4 | 1.40 | 40.6 | 2.80 | 39.7 | 2.40 |
| $B_{12}$ | 2.4 | 1.19 | 0.225 | 1.15 | 0.217 | 1.25 | 0.236 | 1.25 | 0.236 | 1.15 | 0.217 |

Figure S1. Enlarged representation of the diagrams showing the concentrations in µg/L of the B vitamins thiamine (A), riboflavin (B), niacin (C), pantothenic acid (D), pyridoxine (E), biotin (F), folate (G), and cobalamin (H) analyzed in wort as well as in non-alcoholic beers fermented with the yeast strains *S. ludwigii* SL17, *C. saturnus* 247, *C. saturnus* CSa1 and *K. marxianus* 653. Thiamine, riboflavin, niacin and pyridoxine analyses were only conducted for wort and non-alcoholic beers fermented with the yeast strain *S. ludwigii* SL17. Recommended Nutrient Intake (RNI) according to the Food and Agriculture Organization (FAO) and the World Health Organization (WHO), 2001.

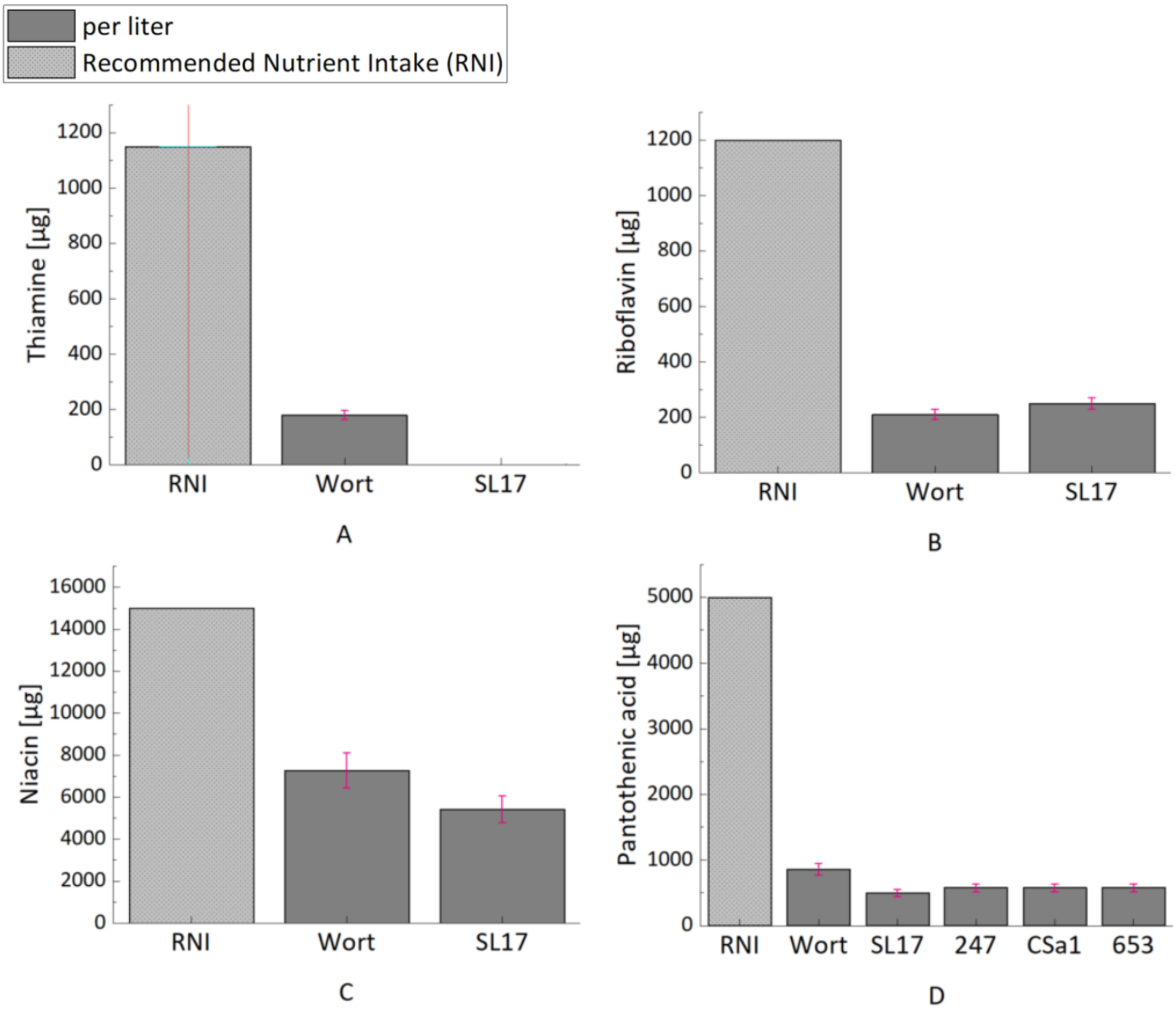

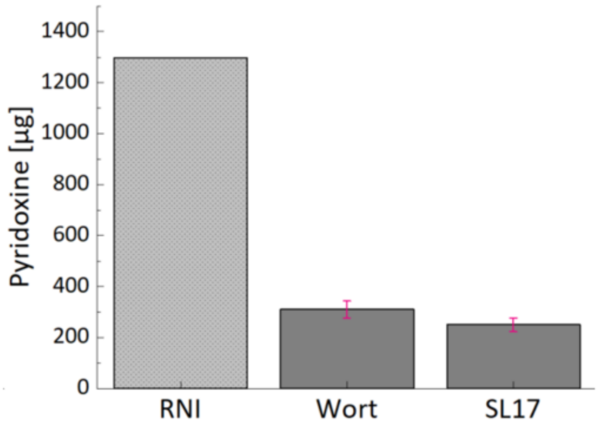


E

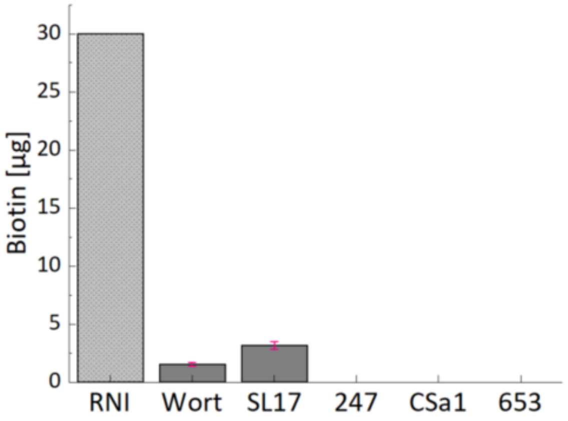


F

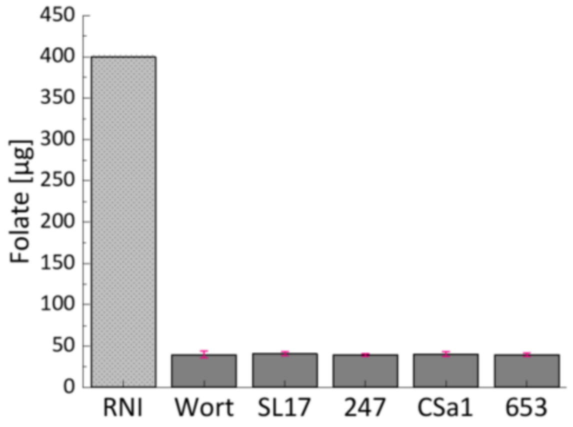


G

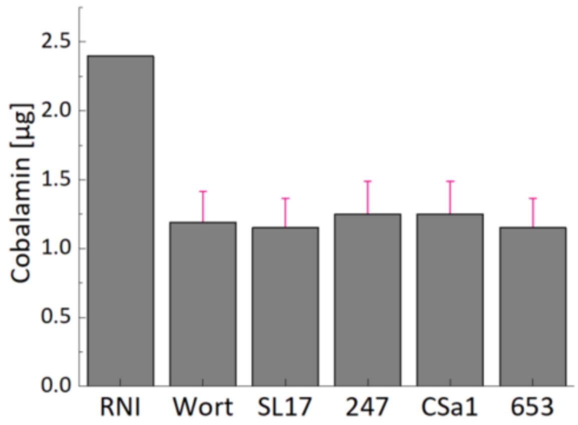


H

Table S3. Analyzed concentrations [µg/g] of the B vitamins thiamine ($B_1$), riboflavin ($B_2$), niacin ($B_3$), pantothenic acid ($B_5$), pyridoxine ($B_6$), biotin ($B_7$), folate ($B_9$) and cobalamin ($B_{12}$) in the propagated yeast *S. ludwigii* S. lud SL17 as well as in the sedimented yeast S. lud SL17. SGS Institut Fresenius GmbH, Freiburg, Germany who performed the analyses is accredited according to DIN EN ISO/IEC 17025. Due to the accreditation, the coefficients of variation (CV) were calculated from the respective standardized methods and were used for the statistical evaluation.

| B Vitamin [µg/g] | Propagated Yeast S. lud SL17 | CV Propagated Yeast S. lud SL17 | Sedimented Yeast S. lud SL17 | CV Sedimented Yeast S. lud SL17 |
|---|---|---|---|---|
| $B_1$ | 18 | 1.62 | 33 | 2.97 |
| $B_2$ | 4.7 | 0.409 | 5.2 | 0.452 |
| $B_3$ | 95 | 11.1 | 100 | 11.7 |
| $B_5$ | 8.6 | 0.877 | 6.9 | 0.704 |
| $B_6$ | 2.7 | 0.289 | 2.9 | 0.310 |
| $B_7$ | 0.60 | 0.061 | 0.42 | 0.043 |
| $B_9$ | 6.4 | 0.299 | 5.6 | 0.299 |
| $B_{12}$ | $< 0.25^{-3}$ | 0 | $< 0.25^{-3}$ | 0 |

Table S4. Mean values (n = 3) of original wort [°P], ethanol % [(*v/v*)], apparent attenuation [%] and pH values of beers fermented with 16 different non-*Saccharomyces* yeast strains and one *S. cerevisiae* reference yeast strain for the determination of biogenic amines.

| Yeast Strain Abbreviation | Original Wort [°P] | Ethanol % [(*v/v*)] | Apparent Attenuation [%] | pH Value |
|---|---|---|---|---|
| C. fab 5640 | 11.77 | 0.74 | 12.1 | 4.83 |
| C. fab 5650 | 11.82 | 0.70 | 11.3 | 4.85 |
| C. mis 238 | 11.75 | 0.57 | 9.4 | 4.74 |
| C. mis CM1 | 11.73 | 0.57 | 9.3 | 4.77 |
| C. sat 247 | 11.75 | 0.74 | 12.0 | 4.97 |
| C. sat 4549 | 11.82 | 0.73 | 11.8 | 5.01 |
| C. sat CSa1 | 11.83 | 0.73 | 11.8 | 5.03 |
| K. ser 3C4 | 11.73 | 0.57 | 9.3 | 4.88 |
| K. lac G9K | 11.79 | 0.92 | 15.0 | 4.94 |
| K. mar 653 | 11.66 | 0.83 | 13.7 | 4.56 |
| L. klu 3082 | 11.69 | 0.98 | 16.0 | 4.72 |
| P. klu PK1 | 11.74 | 0.53 | 8.6 | 4.71 |
| S. cer 68 | 12.01 | 3.79 | 60.0 | 4.58 |
| S. lud SL17 | 11.81 | 0.97 | 15.8 | 4.89 |
| S. fib Lu27 | 11.75 | 0.90 | 14.6 | 4.47 |
| S. pom G10S | 11.78 | 0.76 | 12.4 | 4.78 |
| T. del 1I6 | 11.74 | 0.96 | 15.6 | 4.74 |

Table S5. One-sample *t*-test results with Prob > |*t*|of original wort [°P], ethanol % [(*v/v*)], apparent attenuation [%] and pH values of beers fermented with 16 different non-*Saccharomyces* yeast strains and one *S. cerevisiae* reference yeast strain for the determination of biogenic amines.

| Yeast Strain Abbreviation | Original Wort [°P] | Ethanol % [(*v/v*)] | Apparent Attenuation [%] | pH Value |
|---|---|---|---|---|
| C. fab 5640 | 3.45E-06 | 2.01E-05 | 3.05E-05 | 4.76E-07 |
| C. fab 5650 | 7.95E-08 | 2.25E-05 | 3.50E-05 | 8.96E-06 |
| C. mis 238 | 8.04E-08 | 1.03E-04 | 2.41E-04 | 1.48E-06 |
| C. mis CM1 | 2.50E-06 | 1.35E-04 | 2.70E-04 | 2.34E-05 |
| C. sat 247 | 0 | 2.01E-05 | 7.76E-06 | 5.44E-05 |
| C. sat 4549 | 2.39E-07 | 6.25E-05 | 1.04E-04 | 1.33E-06 |
| C. sat CSa1 | 5.56E-07 | 1.47E-04 | 3.11E-04 | 5.70E-06 |
| K. ser 3C4 | 8.07E-08 | 0 | 0 | 3.27E-06 |
| K. lac G9K | 1.68E-06 | 9.25E-05 | 5.93E-05 | 1.82E-06 |
| K. mar 653 | 5.72E-07 | 2.11E-04 | 3.37E-04 | 1.60E-06 |
| L. klu 3082 | 6.83E-06 | 4.66E-05 | 2.25E-04 | 7.22E-04 |
| P. klu PK1 | 3.22E-07 | 4.01E-05 | 5.96E-05 | 1.50E-06 |
| S. cer 68 | 7.71E-08 | 1.00E-05 | 4.01E-06 | 5.30E-07 |
| S. lud SL17 | 2.39E-07 | 1.17E-05 | 1.79E-05 | 3.25E-06 |
| S. fib Lu27 | 8.04E-08 | 1.36E-05 | 5.19E-06 | 5.55E-07 |
| S. pom G10S | 2.40E-07 | 5.19E-04 | 1.95E-04 | 1.46E-06 |
| T. del 1I6 | 5.64E-07 | 8.38E-05 | 1.37E-05 | 1.04E-05 |

At the level < 0.05 the population mean (n = 3) does not differ significantly from the test mean (0).

Table S6. Biogenic amine concentration [mg/L] of the positive control with limit of determination ≥ 5.0 mg/L.

| Biogenic Amine | Positive Control [mg/L] |
|---|---|
| Histamine | < 5.0 |
| Cadaverine | 7.9 |
| Putrescine | 17.0 |
| Spermidine | < 5.0 |
| Spermine | < 5.0 |
| Tyramine | 41.3 |
| Phenylethylamine | < 5.0 |
| Tryptamine | < 5.0 |
| Isopentylamine | < 5.0 |
